# Depression and anxiety symptoms, health behavior–related personal factors, and tobacco smoking problem: a secondary data analysis

**DOI:** 10.3389/fpubh.2026.1886431

**Published:** 2026-07-16

**Authors:** Fátima Méndez-López, Bárbara Oliván-Blázquez, Marta Domínguez-García, Yolanda López-Del-Hoyo, Verónica Casado-Vicente, Ruth Martí-Lluch, Rosa Magallón-Botaya

**Affiliations:** 1 Department of Physiatry and Nursing, Faculty of Health Sciences, University of Zaragoza, Zaragoza, Spain; 2 Aragonese Primary Care Research Group (GAIAP), Institute for Health Research Aragón (IIS Aragón), Zaragoza, Spain; 3 Network for Research on Chronicity, Primary Care and Health Promotion (RICAPPS), Health Institute Carlos III, Madrid, Spain; 4 Department of Psychology and Sociology, University of Zaragoza, Zaragoza, Spain; 5 Aragonese Healthcare Service (SALUD), Zaragoza, Spain; 6 Mental Health Research in Primary Care Group, Institute for Health Research Aragón (IIS Aragón), Zaragoza, Spain; 7 Parquesol University Health Center, Regional Health Management - Castilla y León Health Service (SACYL), Valladolid, Spain; 8 Vascular Health Research Group of Girona, Institut Universitari per a la Recerca a l'Atenció Primària Jordi Gol i Gurina (IDIAPJGol), Girona, Spain; 9 Institut d'Investigació Biomèdica de Girona (IDIBGI), Girona, Spain; 10 Department of Medicine, Psychiatry and Dermatology, University of Zaragoza, Zaragoza, Spain

**Keywords:** anxiety, depression, personal health factors, smoking, tobacco smoking problem

## Abstract

**Background:**

Smoking is a growing public health problem that has a significant impact on psychosocial functioning and quality of life. The presence of certain personal factors on health-promoting behavior influences mental well-being and less dependence on tobacco in the population.

**Objetive:**

The purpose of this study is to explore the association between tobacco abuse, affective disorders (depression and anxiety) and personal factors related to health behavior. We also aimed to analyze how affective problems can mediate the association between personal factors related to health behavior and tobacco abuse.

**Methods:**

Descriptive, bivariate, multivariate and mediation analyzes of data from 391 participants of 35–74 years old in primary health care centers located in Aragón (Spain) were performed between July 2021 and July 2022. The primary variable was tobacco consumption, evaluated qualitatively (presence or absence) and quantitatively, as consumption measured through the number of cigarettes.

**Results:**

Higher patient activation and health literacy were associated with lower odds of tobacco smoking problem and lower daily cigarette consumption. Conversely, being without a partner and having more severe depressive symptoms were associated with higher odds of tobacco smoking problem and higher daily cigarette consumption. The mediation analyses suggested indirect statistical associations involving depressive symptoms in the relationships between health literacy, patient activation, and tobacco smoking outcomes. However, given the cross-sectional design, these findings should not be interpreted as evidence of causal or temporal mediation.

**Conclusion:**

The severity of depressive and anxiety symptoms and personal factors are related to tobacco smoking problem. These associations may be relevant for informing future preventive strategies aimed at reducing smoking rates in the population. However, longitudinal and intervention studies are needed to clarify the temporal and causal relationships among personal health-related factors, depressive symptoms, and tobacco smoking outcomes.

**Clinical Trial Registration:**

Identifier, ISRCTN12820058. https://doi.org/10.1186/ISRCTN12820058

## Background

Smoking is a growing public health problem with high morbidity and mortality worldwide (every year, over 7 million deaths result from tobacco use) (1). Currently, the World Health Organization (WHO) (1) reports that 22.3% of the world population uses tobacco, being more prevalent in men than in women; specifically, 36.7% of all men and 7.8% of women worldwide. In Europe, 15.3% of the population consumes less than 20 cigarettes a day, and 4.4% consumes 20 or more, specifically in Spain these prevalence of consumption stand at 14.8% and 4 0.9%, respectively (2). Smoking occurs in very different cultural contexts, configuring a global phenomenon of increasing magnitude, particularly smoking rates are higher in low ―and middle―income countries, in some cases reaching a prevalence of 60% (1, 3).

Tobacco consumption can induce nicotine dependence and trigger mental and behavioral disorders, such as depressive and anxiety symptoms (4). Recent Mendelian randomization studies have provided evidence supporting potentially causal effects of smoking on depression risk and have also suggested bidirectional relationships between smoking and affective symptoms (5, 6). Many international organizations have acknowledged that tobacco addiction is a significant health problem (World Health Organization’s International Classification of Diseases (ICD-11) (7), International Classification of Primary Care (ICPC-3) (8) and the American Psychiatric Association’s Diagnostic and Statistical Manual, fourth edition (DSM-V) (9). On the other hand, mental health disorders substantially influence the life quality of 246 million individuals globally who have major depressive disorders and 374 million individuals who have anxiety disorders (10). Also, anxiety disorder (6.7%) and depressive disorder (4.1%) are the most commonly recognized mental health conditions in primary health care (PHC) (11).

These mental diseases are the consequence of complex interactions between psychological, social, and biological variables, and they are often associated with considerable suffering or impairment in personal, family-related, social, educational, vocational, or additional areas of functioning (12). The WHO (13) reports that addictions often occur alongside various mental health disorders, such as depression and anxiety. This is particularly true for more frequent addictions like smoking (14). A recent study of 2000 adults showed how nicotine dependence was statistically associated with higher levels of depression and stress (3). Also, a prospective 10-year survey (adjusted for social and demographic factors and mental disorders) showed that anxiety and depression disorders are associated with a 40–50% risk of starting smoking and a 30–40% likelihood of becoming dependent on nicotine (15). Recent evidence obtained during the COVID-19 pandemic further supports the relationship between psychological distress and tobacco use. Karataş (16) reported that COVID-19-related anxiety was associated with stronger smoking urges, with variations according to demographic characteristics, highlighting the complex interaction between psychological vulnerability, contextual stressors, and smoking behavior.

Moreover, the presence of certain personal factors on health-promoting behavior influences mental well-being and less dependence on tobacco in the population (17). Among these factors, health literacy (18), patient activation (19), personality characteristics (20), resilience (21), self-efficacy (22), self-esteem (23) and sense of coherence (24) stand out as elements that highly influence our physical and emotional health.

Health literacy is defined as the population’s knowledge, motivation and individual capacities to understand and make decisions about their health promotion and management (18). A low level of health literacy is associated with an almost 50% higher probability of being a smoker than those with an adequate level (25). In addition, health literacy improvement has a positive effect on people’s emotional status, with a moderately positive effect on lowering depression and anxiety symptoms (26).

Patient activation refers to an individual’s ability and capacity to manage their health condition and confidence in assuming this responsibility (19). Individuals with high activation levels have a strong self-healing capacity, better health status and lower levels of depression (27). In addition, smokers with increased activation tend to seek advice and are more likely to quit smoking than smokers with low activation (28).

The personality characteristics that affect mental health and addictions are introversion, low conscientiousness, neuroticism, low agreeableness and low openness (20). Individuals with high neuroticism and lower conscientiousness have higher levels of anxiety and depression (29) and a greater risk of smoking (30).

Resilience represents a positive adaptation to circumstances of significant adversity, such as misfortunes and adverse life events (21). People with high resilience are better able to cope with untoward life situations, which can lead to improved overall health outcomes, such as a lower risk of smoking and nicotine dependence (17) and lower levels of depression, hopelessness, anxiety and mortality (31, 32). Additionally, resilience appears to buffer the impact of stress and negative emotions on smoking behavior, suggesting that emotional distress may partially explain the association between resilience and tobacco use (33).

Self-efficacy refers to assurance in one’s ability to manage specific life stressors (22). Individuals with high self-efficacy have better emotional regulation and more effective psychosocial functioning (34, 35), and are more likely to quit smoking (36).

Self-esteem refers to the emotions one holds towards oneself, which can be positive or negative (23). Adults with high global self-esteem are likelier to have greater physical, mental, occupational, and social well-being. Low self-esteem is related to emotional problems, substance abuse, eating disorders, smoking and excessive alcohol consumption (37, 38).

Finally, sense of coherence refers to an individual’s disposition towards the essential values for their overall well-being and life experiences (24). Individuals who possess a strong sense of coherence are more inclined to maintain good mental health and adopt healthy lifestyle habits, which may include reducing or quitting smoking (39, 40). Although these constructs have been studied independently, they share a common underlying feature: they represent personal resources that facilitate adaptive coping, self-management, and health-promoting behaviors. Individuals with greater personal resources tend to experience lower psychological distress and are more capable of adopting and maintaining healthy lifestyles, whereas deficits in these resources may increase vulnerability to depression, anxiety, and maladaptive behaviors such as smoking (24, 41, 42).

Antonovsky’s salutogenic approach is based on these personal factors (41). This theory of health aims to increase the mental health and well-being of participants by improving their knowledge, confidence, and ability to reinforce personal characteristics associated with healthy behavior (42). The relationship of personal characteristics with health is especially relevant in PHC due to its accessibility and interaction with people with mental health problems and their families (43). In addition, the integration of mental health into primary care promotes comprehensive, coordinated and personalized care for many people with comorbid physical and mental health problems (44).

Considering prior evidence, we know of patterns of certain psychological constructs associated with smoking cessation.

Anxiety and depressive symptoms may represent important psychological mechanisms linking individual resources and smoking behavior. Individuals with lower resilience, self-efficacy, self-esteem, or sense of coherence often exhibit greater emotional vulnerability and poorer coping skills when facing stressors. Consequently, smoking may be adopted or maintained as a maladaptive emotion-regulation strategy aimed at reducing negative affect and psychological distress (45). Furthermore, depressive and anxiety symptoms have been associated with stronger withdrawal-related negative affect, increased craving, and greater difficulty achieving smoking cessation, suggesting a potential pathway through which psychological distress contributes to nicotine dependence (46).

Previous evidence suggests that personal psychological resources and affective symptoms may operate within interconnected pathways. For example, self-efficacy has been identified as a mediator of the relationship between depressive symptoms and smoking susceptibility among adolescents, highlighting the close interplay between emotional distress and personal health-related resources in tobacco-related behaviors (47).

However, there is not as much evidence on how mental health (depression and/or anxiety) can influence the relationship between personal factors on health behavior and tobacco smoking problem. Therefore, our study aimed to analyze the association between tobacco abuse, affective disorders (depression and anxiety) and personal factors related to health behavior (resilience, self-efficacy, sense of coherence, patient activation, health literacy, self-esteem and personality characteristics). We hypothesized (H1) that persons with more severe tobacco abuse problems (i.e., excessive tobacco use and dependence) would show greater severity of depressive and anxiety symptoms, as well as lower levels of resilience, self-efficacy, sense of coherence, patient activation, health literacy, self-esteem and personality characteristics than non-smoking persons.

We also aimed to analyze how affective problems (depression and/or anxiety) can mediate the association between personal factors related to health behavior and tobacco abuse. Based on the salutogenic framework, we expected that lower levels of personal health resources would be associated with greater depressive and anxiety symptomatology, which in turn would be related to greater tobacco smoking problem (H2).

## Methods

### Study design

This research project is a secondary data analysis (48) focuses exclusively on baseline data collected at the start of a prospective longitudinal cohort study (49) whose main objective is to analyze the relationship between psychological constructs (self-efficacy, activation, health literacy, resilience, personality traits, sense of coherence, self-esteem), affective-emotional problems (anxiety, depression) and addiction. This study was conducted between July 2021 and July 2022 in PHC centers in Aragón, northern Spain. It was registered with the ISRCTN Registry on 21 March of 2022 (ISRCTN12820058).

### Participants and recruitment

The study population was participants between 35–74 years old, who understood written and spoken Spanish and provided consent to participate in the study voluntarily. The exclusion criteria were having a terminal illness, cognitive dysfunction, dementia, or any significant illness that could seriously interfere with participation in the study. The participants were selected from the lists of the PHC centers of which they were users. To select the cohort participants, a stratified selection was conducted by age, sex, and population distribution in urban and rural areas concerning the data from the Aragon census of the National Institute of Statistics (50). The purpose of this stratification was to provide maximum diversity to the sample while ensuring representativeness of the population, as explained in the study protocols (49, 51). Primary care coverage in our country is practically universal, so almost all the population of the region is also a user of PHC. The study was conducted at two PHC centers (one rural and one urban center). The rural center sample was a town of less than 2000 inhabitants. Following the stratification criteria, individuals were randomly selected from the list of users of the participating health centres. The selected participants were contacted by letter or telephone, where they were fully informed of the study. Those who showed interest in participating voluntarily made an appointment at their usual health centre to complete the questionnaires. The study adhered to the Strengthening the Reporting of Observational studies in Epidemiology (STROBE) guidelines (52).

The sample size was established in the prospective longitudinal cohort study on which this analysis of secondary data is based (49). Its methodology established a necessary sample size of 290 participants, according to the prevalence of the most frequent affective disorders and addictions in primary care (anxiety, depression) as the main variable. Finally, 505 people were evaluated for eligibility (312 participants from the urban center, 193 participants from the rural center). Of them, 105 people did not agree to participate in the study, of which 71 were not interested, and 34 were interested, but reported not having time to participate, finally including 400 participants in the study, considerably exceeding the sample size requirement. The participation rate was 79.16%.

### Outcomes and measures

We collected information on sex (woman, man), age, municipality classification (urban, rural), marital status (without a partner: single, separated, divorced, widower or widow; and with a partner: married or living with a partner), education (none or primary and secondary or tertiary), occupation (working active, not working: unemployment, homemaker, unpaid work, student, pensioner, sick leave, temporary job disability, permanent job disability, and other situations) through an *ad hoc* questionnaire.

The main variable was tobacco consumption, evaluated as a qualitative variable (presence or absence) and quantitatively, as consumption measured through the number of cigarettes. The diagnosis of a tobacco smoking problem (code P14) was assessed on their electronic medical record according to the criteria of the International Classification of Primary Care (ICPC-3). Tobacco abuse is defined as the problem of its use resulting in one or more of the following: harmful use with clinically important damage to health, dependence syndrome and withdrawal state (8). Also, the consumption was examined through an *ad hoc* questionnaire prepared by the research team and adapted from the WHO study (53).

Secondary outcomes were depressive and anxious symptomatology and some related personal factors with health behavior (resilience, health literacy, activation, self-efficacy, sense of coherence, self-esteem, personality characteristics).

The severity of depression was measured using the Patient Health Questionnaire (PHQ-9, Spanish version) (54), which measures the presence and severity of depression. The severity levels included no depression (0–4), mild depression (5–9), moderate depression (10–14), moderately severe depression (15–19) and severe depression (20–27). The validated Spanish version has a Cronbach’s *α* value of 0.80 (55). The internal consistency of PHQ-9 in our sample was 0.83.

The presence and severity of anxiety symptoms was measured using the Generalized Anxiety Disorder (GAD-7, Spanish version) (56). It consists of seven multiple-choice questions, with each response being graded on a scale ranging from 0 to 3. Each item describes one of the typical symptoms of generalized anxiety disorders. The severity levels included no anxiety (0–4), mild anxiety (5–9), moderate anxiety (10–14) and severe anxiety (15–21). The validated Spanish version has a Cronbach’s *α* value of 0.93 (57). GAD-7 had good internal consistency (Cronbach’s *α* = 0.84).

Health literacy was measured using the Health Literacy Survey European Questionnaire (HLS-EU-Q16, Spanish version) (18). Higher scores (after transforming each into a dichotomous response) (range 0–16) indicate a higher level of health literacy: inadequate or problematic health literacy levels (0–12) and sufficient or adequate health literacy (13–16). In its Spanish version, Cronbach’s *α* coefficient is 0.98 (58). The internal consistency in our sample was high (α = 0.87).

Patient activation was measured using the Patient Activation Questionnaire (PAM-13, Spanish version) (19). Higher scores indicate a higher level of patients’ activation in addressing their health (range 0–100) and place the individual at one of four levels of activation: “Disengaged and overwhelmed” (0–47.0), “Becoming aware but still struggling” (47.1–55.1), “Taking action” (55.2–67.0), and “Maintaining behaviors and pushing further” (67.1–100). In its Spanish version, Cronbach’s *α* is 0.7 (59). The internal consistency in our sample was high (*α* = 0.88).

Personality characteristics were assessed using the Big Five Inventory (BFI-10, Spanish version) (20). The questionnaire measures the five factors of personality from the five factors model: High scores of each factor model indicate (1) extraversion, (2) agreeableness, (3) conscientiousness, (4) neuroticism and (5) openness. The validated Spanish version has a Cronbach’s *α* value of 0.78 (60). The internal consistency of our sample was acceptable (*α* = 0.72).

Resilience was measured using the Connor-Davidson Resilience Scale (CD-RISC-10, Spanish version) (21). This scale score on the questionnaire was the sum of the responses obtained for each item (range 0–40), and the highest scores indicated the highest level of resilience. The validated Spanish version has a Cronbach’s α value of 0.85 (61). The internal consistency of our sample was 0.86.

Self-efficacy was assessed using the General Self-Efficacy Scale (GSES-12, Spanish version) (22). The final score on the questionnaire was the sum of the responses obtained on each item (range 12–60), and the highest scores indicated the highest level of self-efficacy. It measures three factors: (1) initiative, (2) effort, and (3) persistence. The validated Spanish version has a Cronbach’s alpha value of 0.69 (62). The internal consistency in our sample was acceptable (*α* = 0.75).

Self-esteem was measured using the Rosenberg Self-Esteem Scale (RSES, Spanish version) (63). The scale ranges from 0 to 30. Scores between 15 and 25 are within normal range; scores below 15 suggest low self-esteem. The validated Spanish version has a Cronbach’s *α* of 0.85 (64). The internal consistency in our sample was acceptable (*α* = 0.79).

The sense of coherence was measured using the Sense of Coherence Scale (SOC-13, Spanish version) (24). It measures the sense of coherence, comprehensibility, manageability and meaningfulness. Higher scores (after reversal of the inverted items) (range 13–91) indicate a greater sense of coherence. The validated Spanish version has a Cronbach’s α of 0.83 (65). The internal consistency in our sample was acceptable (*α* = 0.75).

### Statistical analysis

The normal distribution of the results was verified using the Kolmogorov–Smirnov test. A descriptive analysis (frequencies (*n*) and percentages (%) for categorical variables; mean (M) and standard deviation (SD) for continuous variables) was performed to determine the characteristics of the sample. Secondly, bivariate analyses were performed using the chi-squared test for qualitative variables and the Student’s t-test for continuous variables, with the aim of comparing the different variables between people with tobacco smoking and those without this addition. Thirdly, two types of regression were applied to evaluate the association of the independent variables with tobacco smoking problem (binary multivariate logistic regression for the consumption measured through the diagnosis of smoking and multiple linear regression for tobacco consumption measured by the number of cigarettes consumed daily). Odds ratios (ORs) were calculated with a 95% confidence interval (95% CI) for logistic regression. In addition, different regression models were performed and multicollinearity (VIF and tolerance values) was analyzed to control the influence of possible confounding variables (sociodemographic) and interactions between the independent variables (mental health and personal factors).

**Figure 1:**
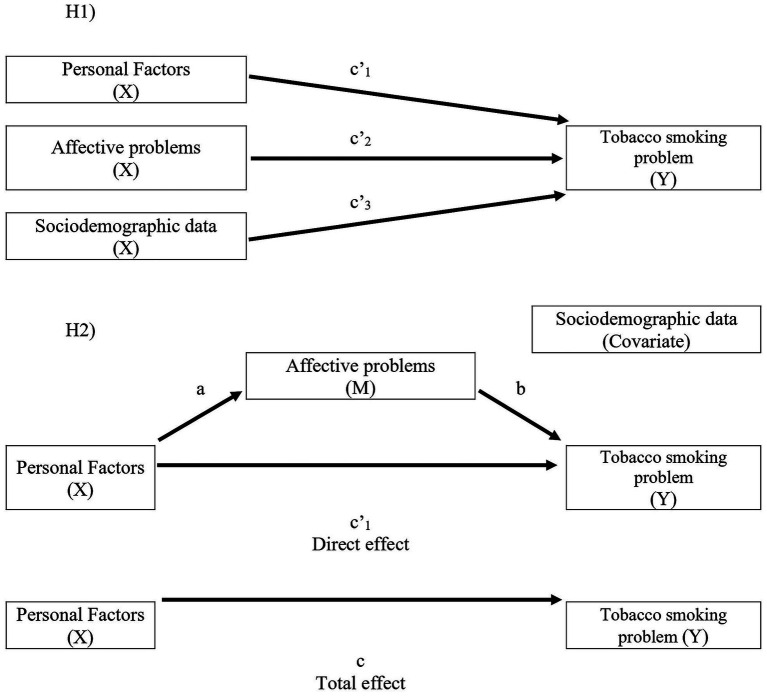
Conceptual models of the hypothesis proposed (H1) hypothesis 1; (H2) hypothesis 2: X: Predictor variable; M: Mediator; Y: outcome variable; a: association of X on M; b: association of M on Y; c’: direct association (without mediator) of X on Y; c: the total effect of X on Y(with affective problems as mediator); ab: indirect association of X on Y.

Finally, several hierarchical multiple regression analyses were conducted to test whether depressive or anxious symptoms mediated the relationship between the personal factors on health-promoting behavior and tobacco smoking problem. Bootstrap resampling (10,000 samples) was used to estimate 95% confidence intervals. Since heteroscedasticity is common in cross-sectional data, all analyses included a correction for it (HC0) (66). The Johnson-Neyman technique was used to compute the range of significance and simple slopes for the interaction analyses (67). We reported unstandardised regression coefficients; all calculations were two-tailed and used conventional significance thresholds (*α* = 0.05). All analyses were performed using IBM SPSS Statistics software (v. 25.0) (68) and mediation analyses were performed using Hayes’s PROCESS macro (v. 4.2) (67). In addition, the theoretical models shown in Figure 1 are conceptually represented to visualize the different associations between variables proposed in our hypothesis. The proposed mediation models were specified *a priori* based on the study protocol and existing theoretical evidence (49).

## Results

A total of 505 people met the inclusion criteria, of which 400 participants agreed to participate and were included in the cohort. Due to the lack of data from the self-administered questionnaires, 9 participants were removed from the final analysis (See Flow diagram in Supplementary Figure S1). Eventually, 391 adults took part (228 women and 163 men). The average age of the sample was 54.57 (SD = 10.72), with ages ranging from 35 to 74 years. Of the 391 participants, 15.85% had tobacco smoking problem (*n* = 62). Table 1 shows sociodemographic and personal factors variables.

**Table 1:** Sociodemographic, psychological and personal characteristics of the participants.

Variables	Total sample (*n* = 391)	Tobacco smoking problem (*n* = 62)	Without Tobacco smoking problem (*n* = 329)	*p*-value
Sex, *n* (%)				
Woman	228 (58.31)	36 (58.06)	192 (58.35)	0.966
Man	163 (41.69)	26 (41.93)	137 (41.64)	
Age, years*, M ± SD*	54.57 ± 10.72	53.52 ± 8.80	54.77 ± 11.04	0.398
Municipality classification, *n* (%)				
Urban	246 (62.92)	41 (66.13)	205 (62.31)	0.568
Rural	145 (37.08)	21 (33.87)	124 (37.68)	
Marital status, *n* (%)				
With a partner	298 (76.21)	41 (66.12)	257 (78.11)	**0.042**
Without a partner	93 (23.79)	21 (33.87)	72 (21.88)	
Occupation, *n* (%)				
Working	243 (62.15)	43 (69.35)	200 (60.79)	0.202
Not working	148 (37.85)	19 (30.64)	129 (39.20)	
Education, *n* (%)				
Secondary or tertiary	292 (74.68)	48 (77.41)	244 (74.16)	0.589
None or primary	99 (25.32)	14 (22.58)	85 (25.83)	
Depression (PHQ-9), M ± SD	2.90 ± 4.09	4.39 ± 2.92	2.61 ± 3.59	**0.002**
Anxiety (GAD-7), M ± SD	2.95 ± 3.79	3.65 ± 3.90	3.76 ± 2.82	0.118
Self-efficacy (GSES-12), M ± SD	45.42 ± 5.90	44.37 ± 6.66	45.62 ± 5.74	0.127
Resilience (CD-RISC-10), M ± SD	27.76 ± 6.81	27.48 ± 5.25	27.81 ± 7.08	0.724
Patient Activation (PAM-13), M ± SD	62.88 ± 15.92	57.34 ± 13.50	63.92 ± 7.08	**0.003**
Health Literacy (HLS-EU-Q16), M ± SD	14.10 ± 2.06	13.43 ± 2.16	14.22 ± 2.02	**0.006**
Personality (BFI-10), M ± SD				
Extraversion	5.24 ± 1.79	5.19 ± 1.915	5.24 ± 1.77	0.842
Agreeableness	6.69 ± 1.80	6.74 ± 1.83	6.69 ± 1.79	0.835
Conscientiousness	4.42 ± 1.59	4.66 ± 1.47	4.38 ± 1.62	0.209
Neuroticism	6.10 ± 2.10	5.62 ± 2.12	6.19 ± 2.08	0.053
Openness	5.13 ± 1.63	5.30 ± 1.62	5.12 ± 1.63	0.421
Sense of coherence (SOC-13), M ± SD	57.04 ± 6.74	55.75 ± 6.79	57.28 ± 6.72	0.102
Self-esteem (RSES score), M ± SD	33.81 ± 3.94	32.88 ± 4.60	33.98 ± 3.79	**0.045**

Statistically significant values (*p* ≤ 0.05) are printed in bold. BFI-10, Big Five Personality Inventory; CD-RISC-10, Connor-Davidson Resilience Scale; GAD-7, Generalized Anxiety Disorder; GSES-12, General Self-Efficacy Scale; HLS-EU-Q16: Health Literacy Survey European Questionnaire; M, mean; n, frequencies; %, percentages; PAM-13: Patient Activation Questionnaire; PHQ-9, Patient Health Questionnaire; RSES, Rosenberg Self-Esteem Scale; SD, standard deviation; SOC-13, Sense of Coherence Questionnaire.

### Association between smoking addition, mental health and personal factors

Returning to our Hypothesis 1 of Figure 1, we developed different multivariate logistic regression models to test whether the diagnosis of smoking was associated with the sociodemographic data, mental health and personal factors (Table 2).

**Table 2:** Regression models of tobacco smoking problem diagnosis, sociodemographic data, mental health and personal factors.

Variables	Model A		Model B	
	Nagelkerke’s R^2^ = 0.03; *p*-value = 0.361		Nagelkerke’s R^2^ = 0.167; *p*-value = 0.005	
	Odds Ratio (95% CI)	*p*-value	Odds Ratio (95% CI)	*p*-value
Sex (Man)	1.022 0.583, 1.791	0.940	1.075 0.562–2.057	0.828
Age	0.996 0.963, 1.030	0.800	0.993 0.956–1.031	0.703
Municipality (urban)	0.762 0.418, 1.386	0.373	0.694 0.333–1.457	0.410
Marital status (without a partner)	**1.942 1.065, 3.538**	**0.030**	1.673 0.855–3.274	0.133
Occupation (not working)	0.697 0.333, 1.457	0.337	0.460 0.202–1.049	0.065
Education (none or primary)	1.025 0.499, 2.107	0.947	1.196 0.531–2.693	0.666
Depression (PHQ-9)			**1.814 1.111–2.961**	**0.017**
Anxiety (GAD-7)			0.938 0.841, 1.045	0.245
Self-efficacy (GSES-12)			0.989 0.928, 1.053	0.723
Resilience (CD-RISC-10)			0.992 0.982, 1.116	0.159
Patient Activation (PAM-13)			**0.980 0.960–0.999**	**0.047**
Health Literacy (HLS-EU-Q16)			**0.794 0.670–0.941**	**0.008**
Extraversion (BFI-10)			0.900 0.750, 1.081	0.262
Agreeableness (BFI-10)			0.990 0.827, 1.184	0.912
Conscientiousness (BFI-10)			0.973 0.853, 1.241	0.768
Neuroticism (BFI-10)			0.909 0.777, 1.064	0.236
Openness (BFI-10)			1.086 0.889, 1.325	0.419
Sense of coherence (SOC-13)			0.974 0.928, 1.023	0.296
Self-esteem (RSES)			0.967 0.886, 1.056	0.459

Statistically significant values (*p* ≤ 0.05) are printed in bold. BFI-10, Big Five Personality Inventory; CD-RISC-10, Connor-Davidson Resilience Scale; CI, confidence interval; GAD-7, Generalized Anxiety Disorder; GSES-12, General Self-Efficacy Scale; HLS-EU-Q16: Health Literacy Survey European Questionnaire; OR: Odds Ratio; PAM-13: Patient Activation Questionnaire; PHQ-9, Patient Health Questionnaire; RSES, Rosenberg Self-Esteem Scale; SD, standard deviation; SOC-13, Sense of Coherence Questionnaire.

First, to evaluate the possible confounding variables, we carried out a model in which we only analyzed the sociodemographic data and their influence in the smoking diagnosis (Model A). In this model A, we find that being without a partner was a predictor of having more risk for the diagnosis of smoking (OR 1.94 *p* = 0.030). Second, in model B we include the three components of this research: sociodemographic data, personal factors, and affective disorders. In this model we found that people who have high patient activation (OR 0.980 *p* = 0.047) and high levels of health literacy (OR 0.794 *p* = 0.008) had a lower risk of being smokers. However, people with more depressive symptoms were independently associated with tobacco smoking problem (OR 1.814 *p* = 0.017). This model explains 16.7% of the possibilities of characterizing smoking (*p* = 0.005).

### Association between daily tobacco consumption, mental health and personal factors

Following our Hypothesis 1, and as we already commented on the methodology, we have analyzed tobacco consumption as a qualitative variable (presence or absence) and quantitatively, as consumption measured through the number of cigarettes. To test whether quantitative daily smoking is associated with mental health and personal factors, different multivariate linear regression models were performed (Table 3).

**Table 3:** Regression models of tobacco consumption, sociodemographic data, mental health and personal factors as variables associated.

Variables	Model A				Model B			
	R2 adjusted = 0.021; *p* = 0.228				R2 adjusted = 0.065; *p* < 0.001			
	*β*	*t* value	*p-*value	Tolerance (VIF)	*β*	*t* value	*p*-value	Tolerance (VIF)
Sex (Man)	0.043	0.845	0.399	0.984 (1.016)	0.046	0.854	0.394	0.849 (1.117)
Age	0.017	0.264	0.792	0.587 (1.705)	0.013	0.202	0.840	0.549 (1.182)
Municipality (urban)	−0.024	−0.445	0.649	0.939 (1.065)	−0.023	−0.408	0.660	0.756 (1.323)
Marital status (without a partner)	**0.124**	**2.436**	**0.015**	**0.977 (1.023)**	0.094	1.818	0.070	0.910 (1.098)
Occupation (not working)	0.075	1.170	0.243	0.619 (1.614)	0.128	1.878	0.059	0.679 (1.320)
Education (none or primary)	0.029	0.509	0.611	0.807 (1.240)	0.022	0.380	0.704	0.741 (1.349)
Depression (PHQ-9)					**0.195**	**2.709**	**0.007**	**0.469 (2.131)**
Anxiety (GAD-7)					−0.115	−1.626	0.105	0.482 (2.076)
Self-efficacy (GSES-12)					−0.025	−0.408	0.684	0.636 (1.571)
Resilience (CD-RISC-10)					−0.046	−0.760	0.448	0.657 (1.521)
Patient activation (PAM-13)					**−0.121**	**−2.279**	**0.023**	**0.864 (1.158)**
Health literacy (HLS-EU-Q16)					**−0.141**	**−2.307**	**0.022**	**0.652 (1.534)**
Extraversion (BFI-10)					−0.020	−0.381	0.704	0.849 (1.178)
Agreeableness (BFI-10)					−0.008	−0.160	0.873	0.893 (1.119)
Conscientiousness (BFI-10)					−0.003	−0.061	0.951	0.935 (1.070)
Neuroticism (BFI-10)					−0.037	−0.656	0.512	0.781 (1.280)
Openness (BFI-10)					0.061	1.146	0.253	0.853 (1.172)
Sense of coherence (SOC-13)					−0.087	−1.557	0.120	0.774 (1.292)
Self-esteem (RSES)					−0.026	−0.439	0.661	0.680 (1.470)

Statistically significant values (*p* ≤ 0.05) are printed in bold. Dependent variable: number of cigarettes per day. B, unstandardized regression coefficient; *β*, standardized regression coefficient; BFI-10, Big Five Personality Inventory; CI, confidence interval; GAD-7, Generalized Anxiety Disorder; GSES-12, General Self-Efficacy Scale; HLS-EU-Q16: Health Literacy Survey European Questionnaire; PAM-13: Patient Activation Questionnaire; PHQ-9, Patient Health Questionnaire RSES, Rosenberg Self-Esteem Scale; SE, standard error; SOC-13, Sense of Coherence Questionnaire; VIF, variance inflation factor.

First, we evaluated the sociodemographic data as possible confounding variables in model A, and we founded that being without a partner was a predictor of higher daily cigarette consumption (*β* = 0.124; *p* = 0.015). Secondly, we developed Model B, in which we controlled for the possible influence of sociodemographic variables, including all the variables. In this model we found that low activation (*β* = − 0.121; *p* = 0.023), low health literacy (*β* = −0.141; *p* = 0.022), and high level of depression (*β* = 0.195; *p* = 0.007), are factors associated with of increased daily tobacco use. This model explains 6.5% of the overall variance (R2 adjusted = 0.065, *F*(3,387) = 8.895, *p* < 0.001) (Table 3).

### The mediating role of mental health on personal factors and tobacco smoking problem

**Figure 2:**
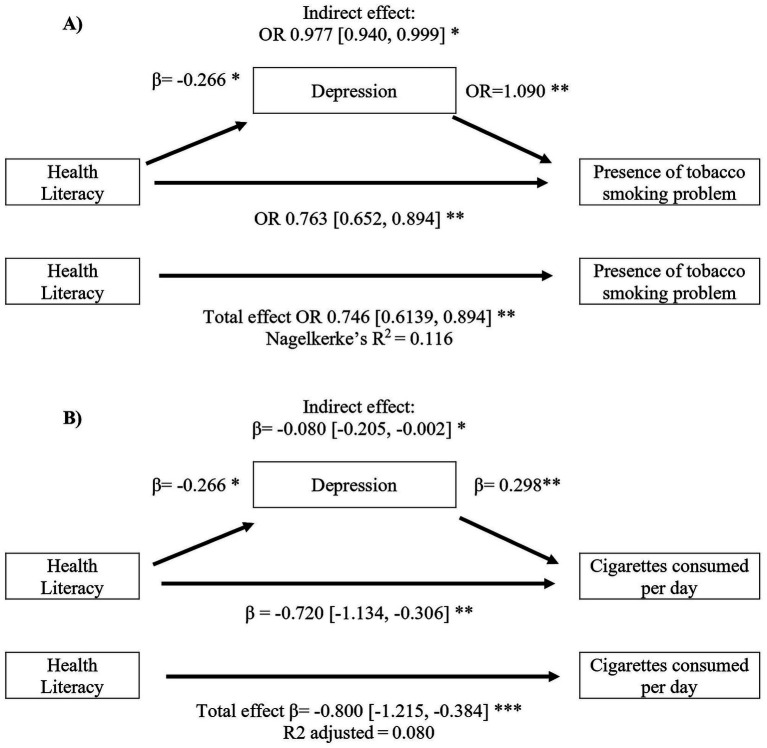
Mediation analysis models. The mediating role of depression between health literacy and **(A)** the presence of *tobacco smoking problem*; **(B)** cigarettes consumed per day. Statistically significant values (*p* ≤ 0.05) are printed in bold. *β*, standardized regression coefficient; OR, Odds Ratio; R2, total variance.

**Figure 3:**
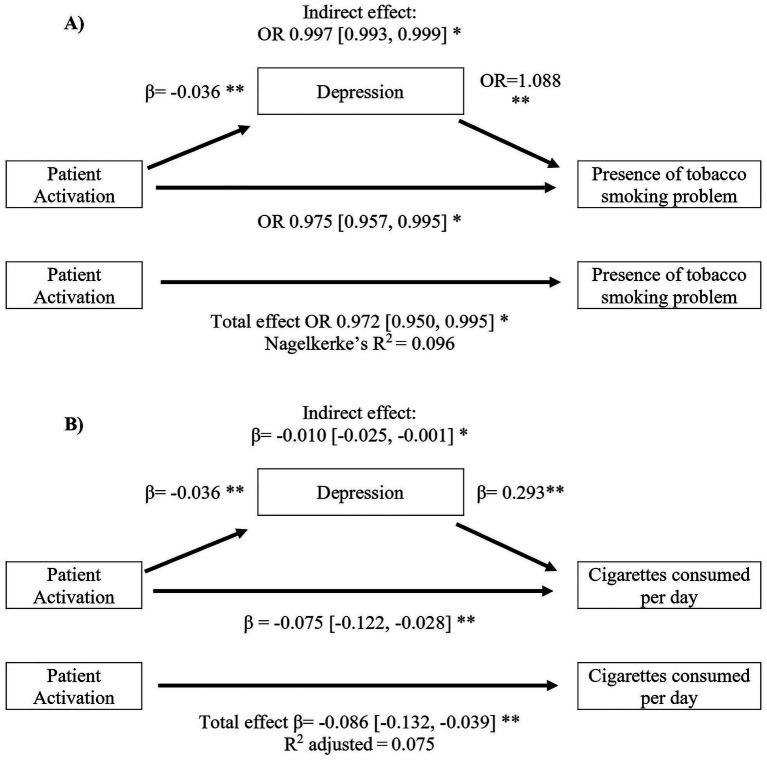
Mediation analysis models. The mediating role of depression between patient activation and **(A)** the presence of *tobacco smoking problem*; **(B)** cigarettes consumed per day. Statistically significant values (*p* ≤ 0.05) are printed in bold. *β*, standardized regression coefficient; OR, Odds Ratio; R2, total variance; Statistically significant values are represented as *p < 0.05; **p < 0.01; ***p < 0.001.

Regarding our Hypothesis 2, we analyzed how affective problems (depression and/or anxiety) can mediate the association between personal factors related to health behavior and tobacco abuse. Given the cross-sectional nature of the data, these mediation analyses should be interpreted as cross-sectional indirect statistical associations rather than evidence of causal mechanisms. Only the associations mediated through depression were significant, specifically with the psychological constructs of health literacy (Figure 2) and patient activation (Figure 3), both for the presence of a smoking problem and the number of cigarettes consumed daily. The other associations mediated by depression and the associations mediated by anxiety were not significant, as can be seen in Supplementary Tables S1–4.

The absence of depressive symptoms improves the relationship between higher levels of health literacy and lower dependence on tobacco (OR 0.746; 95% CI [0.6139–0.894] and lower tobacco consumption (*β* = −0.800; *p* < 0.001) compared to those with more severe depressive symptoms, as shown in Figure 2. Moreover, these absences of depressive symptoms improve the relationship between higher levels of patient activation and lower dependence on tobacco (OR 0.972; 95% CI 0.950–0.995) and lower tobacco consumption (*β* = −0.086; *p* = 0.002) compared to those with more severe depressive symptoms, as shown in Figure 3.

## Discussion

This study analyzed the association between tobacco smoking problem, sociodemographic data, affective disorders (depression and anxiety) and personal factors related to health behavior (resilience, self-efficacy, sense of coherence, patient activation, health literacy, self-esteem, and personality characteristics). Individuals with a smoking habit showed higher levels of depression and lower scores on patient arousal, health literacy, and self-esteem compared to individuals who were non-smokers. These results were statistically significant.

Data from the multivariate analysis demonstrated that a high activation and health literacy were independently associated with having a lower risk of being a smoker as well as a lower daily cigarette consumption, in line with other investigations such as Stewart et al., (69). In contrast, being single and having more severe depressive symptoms were independently associated with tobacco smoking problem and higher daily cigarette consumption.

There are some similar studies in the scientific literature. However, they do not analyze the mediating relationship of depression and anxiety on the association between personal factors and nicotine dependence.

Regarding marital status, in a population of 11,889 adults over 30 years of age in the US, Ramsey et al., (70) observed that adults who do not have a partner are among the most frequent smokers. No other personal conditions have been found as a predictor of smoking.

Activation plays a favorable role in smoking as observed in the cross-sectional study of 25,047 adults by Greene & Hibbard (71) in which the predicted probability of smoking was one percentage point lower when patient arousal was higher. In addition, the studies highlight how training in improving the patient’s activation plays a favorable role in smoking cessation (72). Regarding health literacy, some research supports the effectiveness of increasing HL to reduce smoking, thus demonstrating that, as in our case, higher HL levels are associated with less tobacco dependence in samples of university patients (73, 74). In a systematic review of over 66 manuscripts, Li et al., (75). concluded that adequate health literacy can help promote smoking cessation and prevent relapse to smoking. Regarding other personal factors, Szinay et al., (38). analyzed self-esteem and its relationship with smoking in a large sample of adults in the United Kingdom after adjusting for age, sex, socioeconomic status, and depressed mood, finding a significant association between low self-esteem and smoking. In our case, we have also found an association, but self-esteem has not been shown to be a predictor of smoking. Most of the research is focused on smoking cessation interventions and their relationship with personal factors. For example, Al Thani et al., (76) found a significant association between greater self-efficacy and smoking cessation in a sample of daily smoking men in Qatar. We have not analyzed the effect of interventions for smoking cessation, and we only analyzed predictive factors of smoking. In addition, many studies relate personal factors to very specific population samples and not to the general population (77).

There is evidence that tobacco users have high comorbidity with several mental health disorders. Data from the Netherlands Study on Depression and Anxiety demonstrated that in psychiatric patients, smoking is associated with increased severity of depressive and anxiety symptoms (78). Also, in an investigation carried out in the United Kingdom, it was observed that smoking is associated with schizophrenia and depression (6). In fact, in a systematic review, it was noted that approximately 50% of studies reported that prior depression was related to some subsequent smoking behavior; however, more than a third of them found evidence that smoking was associated with later anxiety/depression (4). The possible genetic association between smoking and depression is even being investigated, still without conclusive results, although from a clinical point of view, smokers were more likely to have depression and anxiety than non-smokers (5). Although our research did not show significant values associated with anxiety, other studies have shown that anxiety was identified as a predictor of tobacco use (4, 46). Regarding the results obtained in our study, a recent report highlighted considerable discrepancies about the precise role of anxiety in the initiation, severity, and outcomes of smoking cessation. The smoking population often refers to smoking as an effective method to control their anxiety (45). This may have been reflected in the anxiety scores of our tobacco-dependent population; however, this issue was not assessed in our participants.

Furthermore, our study also examined how affective problems (depression and/or anxiety) can mediate the association between personal factors related to health behavior and tobacco abuse.

Only the associations mediated through depression were significant, specifically with the psychological constructs of health literacy and patient activation, both for the presence of a smoking problem and the number of cigarettes consumed daily. This means that the absence of depressive symptoms improves the relationship between higher levels of health literacy or patient activation, lower dependence on tobacco, and lower tobacco consumption compared to those with more severe depressive symptoms.

We have not found the association among depression, health literacy or patient activation and smoking in other studies. Other studies have investigated these similar personal factors with different results. For example, Wang et al., (33). found in a population sample a mediating effect of depression and anxiety between resilience and smoking. Our divergence is probably due to different tests used in the methodology (different resilience scales, mental health scales, and different ways of evaluating smoking), in addition to including only an urban population while our sample includes both urban and rural populations. On the other hand, Minix et al., (47) found that self-efficacy mediates the association between mental health (anxiety and depression) and the risk of smoking and nicotine dependence. Furthermore, some studies have measured the moderating role of different personal factors in other addictions (79).

In our models, the values of R2 were between 0.065 and 0.167. Although these values may seem statistically low, it should be remembered that people’s health depends on the combination of many more biological, social, and environmental factors than we include in our model, as explained in the model of determinants of health of the Dahlgren & Whitehead model (80). Above all, R2 values of at least 0.100 are good for explanatory or predictive models in those analyses involving social factors (81).

In any case, a clearer knowledge about the interrelation between smoking, personal factors and mental health (depression or anxiety), and the directionality of these relationships would undoubtedly help to prevent and improve smoking cessation programs, together with the in-depth exploration of other addictions. More studies of this type are needed, with larger sample sizes and longitudinal analyses, where there may be greater capacity to detect the association of affective disorders and personal factors in the development of smoking. Based on these findings, future longitudinal and intervention studies should examine whether strategies aimed at improving health literacy and patient activation may contribute to better management of depressive symptoms and lower tobacco smoking rates. However, given the cross-sectional design of the present study, these potential pathways should be considered hypotheses for future research rather than conclusions derived from the current data. Alternative explanations should also be considered. For instance, reverse causality cannot be ruled out, since tobacco smoking may contribute to the development or worsening of depressive symptoms, rather than depressive symptoms necessarily preceding smoking behavior. In addition, residual confounding by unmeasured biological, social, environmental, or lifestyle-related factors may have influenced the observed associations. Although investigating tobacco prevention and cessation programs was beyond the scope of this study, a better understanding of the interrelationships among personal health-related factors, depressive symptoms, and smoking behavior may help inform the design of more effective future interventions.

### Strengths

Based on previous evidence, we now know patterns of certain psychological constructs associated with smoking cessation. However, there are few investigations like ours, in which such a broad set of personal factors is analyzed in the same population (self-efficacy, health literacy, activation, resilience, sense of coherence, self-esteem and personality characteristics), severity of depression and anxiety, and how they affect smoking, particularly in adult population. Furthermore, it should be noted that there is no previous evidence on how depression mediates the relationship between personal factors on health behavior, such as health literacy and patient activation, and tobacco smoking problem.

In addition, the profile of the participants corresponded to the most frequent age profile of depression and anxiety, in which affective illnesses and addictions have a significant impact on people who are usually of age to carry out work, economic and social activities (82).

### Limitations

This study has certain limitations that must be noted. First, the present study focuses exclusively on baseline data because follow-up assessments of the cohort were not yet available at the time the analyses were conducted. Consequently, the objectives of this work were limited to examining associations between tobacco use, affective symptoms, and personal health-related factors at a single time point. Therefore, the observed mediation effects should not be interpreted as evidence of causal mechanisms, but rather as indirect statistical associations between personal health-related factors, depressive symptoms, and tobacco smoking outcomes. Longitudinal analyses addressing temporal and causal relationships are planned for future studies based on subsequent follow-up waves. Prospective studies with larger sample sizes will also be needed to validate our findings (83).

Second, because this was a secondary data analysis, causal inferences cannot be established (48), and the associations identified may be difficult to interpret (84). Due to the exploratory nature of this analysis, no specific sample size estimates or *p*-value adjustments were performed. Therefore, the findings should be interpreted with caution and considered preliminary associations that requ ire further study.

Third, the number of variables included in the multivariable logistic regression model was relatively large in relation to the number of participants with tobacco smoking problem. Since only 62 participants were classified as smokers, the events-per-variable ratio was limited, increasing the potential risk of model overfitting and reduced model stability. Therefore, the multivariable findings should be interpreted as exploratory and hypothesis-generating. Future studies with larger samples and longitudinal data are needed to confirm the robustness and temporal direction of these associations.

Fourth, the geographical scope of the sample should be considered. Participants were recruited from primary health care centers in Aragón, Spain, including one rural and one urban center. Although this recruitment strategy allowed the inclusion of participants from different settings, the findings may not be fully generalizable to other regions, countries, or health care systems.

Fifth, the assessment of tobacco smoking problem was based on clinical diagnosis and reported cigarette consumption, but no validated scale specifically designed to assess nicotine dependence, such as the Fagerström Test for Nicotine Dependence, was administered. Consequently, the study could not evaluate the severity of nicotine dependence using a standardized psychometric instrument.

Sixth, the proportion of men and women following the population census could not be fully achieved, as the percentage of women was higher than estimated in the 2021 census. Current research suggests that willingness to participate in research is significantly affected by sex, with women being more likely than men to contribute (85).

Seventh, the survey was conducted using two types of questionnaires, self-administered and hetero-administered, each with its own constraints. Self-administered questionnaires may introduce bias due to missing or misinterpreted questions, whereas hetero-administered questionnaires may increase the risk of social desirability bias or make participants feel monitored by the interviewer (86, 87).

Finally, depression and anxiety symptoms were recorded only once. Therefore, changes in the severity of these affective symptoms, their cumulative burden, and their time-varying relationships with tobacco smoking outcomes were not monitored (45). In addition, the study did not assess whether participants used smoking as a strategy to relieve stress or anxiety, which could have influenced the anxiety scores observed among participants with tobacco smoking problem.

## Conclusion

These findings contribute to the search for a relationship between the characteristics of the individuals, addictions such as smoking, and the presence of affective symptoms of anxiety and/or depression. Our findings reveal that higher patient activation and health literacy were associated with lower odds of tobacco smoking problem and lower daily cigarette consumption. However, being single and having higher depression scores were independently associated with tobacco smoking problem. We also found that depressive symptoms mediate the association between the psychological constructs of health literacy and/or patient activation and the presence of smoking, including a greater number of cigarettes smoked daily. Adults with higher health literacy higher activation, and lower depression scores have a lower risk of smoking habits compared to those with more severe depressive symptoms.

Our findings provide a new approach to smoking prevention by reinforcing personal factors, such as health literacy or motivation. Thus, the educational emphasis on improving people’s health literacy can favor a decrease in tobacco use and better mental health. The study findings provide a foundation for future research on the combined role of personal factors and the severity of affective problems and how they affect addictions, as well as highlighting the need to consolidate this line of research.

## Data Availability

The datasets presented in this study can be accessed in online repositories. https://doi.org/10.5281/zenodo.11100097.

## Glossary

*β*: standardized regression coefficient
BFI-10: big five personality inventory
CD-RISC-10: connor-davidson resilience scale
CI: confidence interval
DSM-5: diagnostic and statistical manual of mental Disorders
EU: European union
GAD-7: generalized anxiety disorder
GSES-12: general self-efficacy scale
HLS-EU-Q16: health literacy survey European questionnaire
ICD-11: international classification of diseases
ICPC-3: international classification of primary care
M: mean
N: frequencies
OR: odds ratio
%: percentages
P: *p*-value
PAM-13: patient activation questionnaire
PHC: primary health care
PHQ-9: patient health questionnaire
R2: total variance
RSES: rosenberg self-esteem scale
SD: standard deviation
SOC-13: sense of coherence questionnaire
STROBE: strengthening the reporting of observational studies in epidemiology.

## References

[1] WHO Tobacco (2022) Available online at: https://www.who.int/news-room/fact-sheets/detail/tobacco (Accessed July 10, 2023)

[2] European Union Eurostat statistics data browser (2023) Available online at: https://ec.europa.eu/eurostat/databrowser/view/ISOC_CI_IFP_FU__custom_5093896/default/table?lang=en (Accessed February 25, 2023)

[3] El-Sherbiny NA Elsary AY. Smoking and nicotine dependence in relation to depression, anxiety, and stress in Egyptian adults: a cross-sectional study. J Family Community Med. (2022) 29:8–16. doi: 10.4103/jfcm.jfcm_290_21, 35197723 PMC8802724

[4] Fluharty M Taylor AE Grabski M Munafò MR. The Association of cigarette smoking with depression and anxiety: a systematic review. Nicotine Tob Res. (2017) 19:3–13. doi: 10.1093/ntr/ntw140, 27199385 PMC5157710

[5] Taylor AE Fluharty ME Bjørngaard JH Gabrielsen ME Skorpen F Marioni RE . Investigating the possible causal association of smoking with depression and anxiety using Mendelian randomisation meta-analysis: the CARTA consortium. BMJ Open. (2014) 4:e006141. doi: 10.1136/bmjopen-2014-006141, 25293386 PMC4187451

[6] Wootton RE Richmond RC Stuijfzand BG Lawn RB Sallis HM Taylor GMJ . Evidence for causal effects of lifetime smoking on risk for depression and schizophrenia: a Mendelian randomisation study. Psychol Med. (2020) 50:2435–43. doi: 10.1017/S0033291719002678, 31689377 PMC7610182

[7] WHO International Statistical Classification of Diseases and Related Health Problems (ICD-11) Geneva (2019) Available online at: https://icd.who.int/en (Accessed February 24, 2023)

[8] World Organization of Family Doctors. In: Boven K Napel H, editors. International Classification of Primary Care (ICPC-3), 3rd Edn. Boca Raton, FL: CRC Press (2022). p. 1–389.

[9] American Psychiatric Association. Diagnostic and Statistical Manual of Mental Disorders (DSM-V-TR). Fifth, Text revision. Washington, DC: American Psychiatric Association Publishing (2022).

[10] Santomauro DF Mantilla Herrera AM Shadid J Zheng P Ashbaugh C Pigott DM . Global prevalence and burden of depressive and anxiety disorders in 204 countries and territories in 2020 due to the COVID-19 pandemic. Lancet. (2021) 398:1700–12. doi: 10.1016/S0140-6736(21)02143-7, 34634250 PMC8500697

[11] Subdirección General de Información Sanitaria; Subdirección General de Información Sanitaria Salud mental en datos: prevalencia de los problemas de salud y consumo de psicofármacos y fármacos relacionados a partir de registros clínicos de atención primaria. BDCAP Series 2. Madrid (2021) Available online at: https://www.sanidad.gob.es/estadEstudios/estadisticas/estadisticas/estMinisterio/SIAP/home.htm (Accessed April 15, 2023)

[12] Sekhon S Gupta V. Mood Disorder Treasure Island (FL): StatPearls Publishing (2022) Available online at: http://www.ncbi.nlm.nih.gov/pubmed/32644337 32644337

[13] WHO. Comprehensive Mental Health Action Plan 2013–2030. Geneva (2021) Available online at: https://www.who.int/publications/i/item/9789240031029

[14] WHO The Vicious cycle of Tobacco use and mental Illness – a double Burden on Health (2021) Available online at: https://www.who.int/europe/news/item/08-11-2021-the-vicious-cycle-of-tobacco-use-and-mental-illness-a-double-burden-on-health (Accessed July 10, 2023)

[15] Swendsen J Conway KP Degenhardt L Glantz M Jin R Merikangas KR . Mental disorders as risk factors for substance use, abuse and dependence: results from the 10-year follow-up of the National Comorbidity Survey. Addiction. (2010) 105:1117–28. doi: 10.1111/j.1360-0443.2010.02902.x, 20331554 PMC2910819

[16] Karatas A. The effect of COVID-19 anxiety and awareness on smoking urges: the moderator effect of demographic factors. J Subst Use. (2023) 28:995–1003. doi: 10.1080/14659891.2023.2250849

[17] Lakshmi R Romate J Rajkumar E George AJ Wajid M. Factors influencing tobacco use behaviour initiation – from the perspective of the capability, opportunity, motivation- behaviour (COM-B) model. Heliyon. (2023) 9:e16385. doi: 10.1016/j.heliyon.2023.e16385, 37292260 PMC10245169

[18] Sørensen K Pelikan JM Röthlin F Ganahl K Slonska Z Doyle G . Health literacy in Europe: comparative results of the European health literacy survey (HLS-EU). European J Public Health. (2015) 25:1053–8. doi: 10.1093/eurpub/ckv043, 25843827 PMC4668324

[19] Hibbard JH Mahoney ER Stockard J Tusler M. Development and testing of a short form of the patient activation measure. Health Serv Res. (2005) 40:1918–30. doi: 10.1111/j.1475-6773.2005.00438.x, 16336556 PMC1361231

[20] John OP Donahue E Kentle R The Big Five Inventory--Versions 4a and 54 Berkeley University of California,Berkeley, Institute of Personality and Social Research (1991) Available online at: https://www.ocf.berkeley.edu/~johnlab/bfi.htm

[21] Campbell-Sills L Stein MB. Psychometric analysis and refinement of the Connor-Davidson resilience scale (CD-RISC): validation of a 10-item measure of resilience. J Trauma Stress. (2007) 20:1019–28. doi: 10.1002/jts.20271, 18157881

[22] Sherer M Maddux JE Mercandante B Prentice-Dunn S Jacobs B Rogers RW. The self-efficacy scale: construction and validation. Psychol Rep. (1982) 51:663–71. doi: 10.2466/pr0.1982.51.2.663

[23] Bailey JA. The foundation of self-esteem. J Natl Med Assoc. (2003) 95:388–93. 12793795 PMC2594522

[24] Antonovsky A. The structure and properties of the sense of coherence scale. Soc Sci Med. (1993) 36:725–33. doi: 10.1016/0277-9536(93)90033-Z, 8480217

[25] Fawns-Ritchie C Starr JM Deary IJ. Health literacy, cognitive ability and smoking: a cross-sectional analysis of the English longitudinal study of ageing. BMJ Open. (2018) 8:e023929. doi: 10.1136/bmjopen-2018-023929, 30368451 PMC6224719

[26] Magallón-Botaya R Méndez-López F Oliván-Blázquez B Carlos Silva-Aycaguer L Lerma-Irureta D Bartolomé-Moreno C. Effectiveness of health literacy interventions on anxious and depressive symptomatology in primary health care: a systematic review and meta-analysis. Front Public Health. (2023) 11:1007238. doi: 10.3389/fpubh.2023.1007238, 36844856 PMC9948257

[27] Blakemore A Hann M Howells K Panagioti M Sidaway M Reeves D . Patient activation in older people with long-term conditions and multimorbidity: correlates and change in a cohort study in the United Kingdom. BMC Health Serv Res. (2016) 16:582. doi: 10.1186/s12913-016-1843-2, 27756341 PMC5069882

[28] Cunningham P. Patient engagement during medical visits and smoking cessation counseling. JAMA Intern Med. (2014) 174:1291–8. doi: 10.1001/jamainternmed.2014.2170, 24911033

[29] Jourdy R Petot J-M. Relationships between personality traits and depression in the light of the “big five” and their different facets. Evol Psychiatr. (2017) 82:e27–37. doi: 10.1016/j.evopsy.2017.08.002

[30] Choi J-S Payne TJ Ma JZ Li MD. Relationship between personality traits and nicotine dependence in male and female smokers of African-American and European-American samples. Front Psych. (2017) 8:122. doi: 10.3389/fpsyt.2017.00122, 28769824 PMC5513910

[31] Smith JL Hollinger-Smith L. Savoring, resilience, and psychological well-being in older adults. Aging Ment Health. (2015) 19:192–200. doi: 10.1080/13607863.2014.986647, 25471325

[32] To QG Vandelanotte C Cope K Khalesi S Williams SL Alley SJ . The association of resilience with depression, anxiety, stress and physical activity during the COVID-19 pandemic. BMC Public Health. (2022) 22:1–8. doi: 10.1186/S12889-022-12911-9/TABLES/4 35279118 PMC8917786

[33] Wang Y Chen X Gong J Yan Y. Relationships between stress, negative emotions, resilience, and smoking: testing a moderated mediation model. Subst Use Misuse. (2016) 51:427–38. doi: 10.3109/10826084.2015.1110176, 26894428 PMC4855524

[34] Milanovic M Ayukawa E Usyatynsky A Holshausen K Bowie CR. Self efficacy in depression. J Nerv Ment Dis. (2018) 206:350–5. doi: 10.1097/NMD.0000000000000804, 29538054

[35] Schönfeld P Preusser F Margraf J. Costs and benefits of self-efficacy: differences of the stress response and clinical implications. Neurosci Biobehav Rev. (2017) 75:40–52. doi: 10.1016/J.NEUBIOREV.2017.01.031, 28143761

[36] de Hoog N Bolman C Berndt N Kers E Mudde A de Vries H . Smoking cessation in cardiac patients: the influence of action plans, coping plans and self-efficacy on quitting smoking. Health Educ Res. (2016) 31:350–62. doi: 10.1093/her/cyv100, 26827369

[37] Orth U Robins RW Widaman KF. Life-span development of self-esteem and its effects on important life outcomes. J Pers Soc Psychol. (2012) 102:1271–88. doi: 10.1037/a0025558, 21942279

[38] Szinay D Tombor I Garnett C Boyt N West R. Associations between self-esteem and smoking and excessive alcohol consumption in the UK: a cross-sectional study using the BBC UK lab database. Addict Behav Rep. (2019) 10:100229. doi: 10.1016/j.abrep.2019.100229, 31720364 PMC6838740

[39] Vyas D Patel M Sharma A Chhabra K Gupta A Mundra R. Impact of self-efficacy and sense of coherence on tobacco cessation motivation and readiness among slum dwellers in Ajmer city during COVID-19 health emergency. J Family Med Prim Care. (2022) 11:1867–75. doi: 10.4103/jfmpc.jfmpc_1821_21, 35800543 PMC9254856

[40] Giglio RE Rodriguez-Blazquez C de Pedro-Cuesta J Forjaz MJ. Sense of coherence and health of community-dwelling older adults in Spain. Int Psychogeriatr. (2015) 27:621–8. doi: 10.1017/S1041610214002440, 25420753

[41] Lindstrom B. Salutogenesis. J Epidemiol Community Health. (1978) 59:440–2. doi: 10.1136/jech.2005.034777, 15911636 PMC1757059

[42] Mittelmark MB Sagy S Eriksson M Bauer GF Pelikan JM Lindström B , editors. The Handbook of Salutogenesis. Cham: Springer International Publishing (2017).

[43] Galvez-Llompart AM Valor Gisbert M Perez-Almarcha M Ballester-Gracia I Canete-Nicolas C Reig-Cebria MJ . Impact on mental health care after collaboration between primary care and mental health. Medicina de Familia Semergen. (2021) 47:385–93. doi: 10.1016/j.semerg.2021.04.006, 34144866

[44] WHO Mental Health in Primary care: Illusion or Inclusion? Geneva: (2018). p. 1–18 Available online at: https://apps.who.int/iris/handle/10665/326298 (Accessed April 16, 2023)

[45] Garey L Olofsson H Garza T Shepherd JM Smit T Zvolensky MJ. The role of anxiety in smoking onset, severity, and cessation-related outcomes: a review of recent literature. Curr Psychiatry Rep. (2020) 22:38. doi: 10.1007/s11920-020-01160-5, 32506166

[46] Leventhal AM Ameringer KJ Osborn E Zvolensky MJ Langdon KJ. Anxiety and depressive symptoms and affective patterns of tobacco withdrawal. Drug Alcohol Depend. (2013) 133:324–9. doi: 10.1016/j.drugalcdep.2013.06.015, 23896304 PMC4049140

[47] Minnix JA Blalock JA Marani S Prokhorov AV Cinciripini PM. Self-efficacy mediates the effect of depression on smoking susceptibility in adolescents. Nicotine Tob Res. (2011) 13:699–705. doi: 10.1093/ntr/ntr061, 21482619 PMC3150689

[48] Wickham RJ. Secondary analysis research. J Adv Pract Oncol. (2019) 10:395–400. doi: 10.6004/jadpro.2019.10.4.7 33343987 PMC7520737

[49] Méndez-López F Oliván-Blázquez B Domínguez-García M Bartolomé-Moreno C Rabanaque I Magallón-Botaya R. Protocol for an observational cohort study on psychological, addictive, lifestyle behavior and highly prevalent affective disorders in primary health care adults. Front Psych. (2023) 14:1121389. doi: 10.3389/fpsyt.2023.1121389, 37363179 PMC10288582

[50] Aragon Statistics Institute Demographic and Population Statistics (2022) Available online at: https://www.aragon.es/-/demografia-y-poblacion (Accessed August 8, 2023)

[51] Martí-Lluch R Bolíbar B Llobera J Maderuelo-Fernández JA Magallón-Botaya R Sánchez-Pérez Á . Role of personal aptitudes as determinants of incident morbidity, lifestyles, quality of life, use of health services, and mortality (DESVELA cohort): quantitative study protocol for a prospective cohort study in a hybrid analysis. Front Public Health. (2023) 11:1067249. doi: 10.3389/fpubh.2023.1067249, 37427254 PMC10325828

[52] von Elm E Altman DG Egger M Pocock SJ Gøtzsche PC Vandenbroucke JP. The strengthening the reporting of observational studies in epidemiology (STROBE) statement: guidelines for reporting observational studies. J Clin Epidemiol. (2008) 61:344–9. doi: 10.1016/j.jclinepi.2007.11.008, 18313558

[53] Molarius A Parsons RW Dobson AJ Evans A Fortmann SP Jamrozik K . Trends in cigarette smoking in 36 populations from the early 1980s to the mid-1990s: findings from the WHO MONICA project. Am J Public Health. (2001) 91:206–12. doi: 10.2105/ajph.91.2.206, 11211628 PMC1446542

[54] Kroenke K Spitzer RL. The PHQ-9: a new depression diagnostic and severity measure. Psychiatr Ann. (2002) 32:509–15. doi: 10.3928/0048-5713-20020901-06

[55] Muñoz-Navarro R Cano-Vindel A Medrano LA Schmitz F Ruiz-Rodríguez P Abellán-Maeso C . Utility of the PHQ-9 to identify major depressive disorder in adult patients in Spanish primary care centres. BMC Psychiatry. (2017) 17:291. doi: 10.1186/s12888-017-1450-8, 28793892 PMC5550940

[56] Spitzer RL Kroenke K Williams JBW Löwe B. A brief measure for assessing generalized anxiety disorder. Arch Intern Med. (2006) 166:1092. doi: 10.1001/archinte.166.10.1092, 16717171

[57] Garcia-Campayo J Zamorano E Ruiz MA Pardo A Perez-Paramo M Lopez-Gomez V . Cultural adaptation into Spanish of the generalized anxiety disorder-7 (GAD-7) scale as a screening tool. Health Qual Life Outcomes. (2010) 8:8. doi: 10.1186/1477-7525-8-8, 20089179 PMC2831043

[58] Nolasco A Barona C Tamayo-Fonseca N Irles MÁ Más R Tuells J . Health literacy: psychometric behaviour of the HLS-EU-Q16 questionnaire. Gac Sanit. (2020) 34:399–402. doi: 10.1016/j.gaceta.2018.08.006, 30473252

[59] Moreno-Chico C González-de Paz L Monforte-Royo C Arrighi E Navarro-Rubio MD Gallart Fernández-Puebla A. Adaptation to European Spanish and psychometric properties of the patient activation measure 13 in patients with chronic diseases. Fam Pract. (2017) 34:627–34. doi: 10.1093/fampra/cmx022, 28379415

[60] Benet-Martínez V John OP. Los Cinco Grandes across cultures and ethnic groups: multitrait-multimethod analyses of the big five in Spanish and English. J Pers Soc Psychol. (1998) 75:729–50. doi: 10.1037/0022-3514.75.3.729, 9781409

[61] Notario-Pacheco B Solera-Martínez M Serrano-Parra MD Bartolomé-Gutiérrez R García-Campayo J Martínez-Vizcaíno V. Reliability and validity of the Spanish version of the 10-item Connor-Davidson resilience scale (10-item CD-RISC) in young adults. Health Qual Life Outcomes. (2011) 9:63. doi: 10.1186/1477-7525-9-63, 21819555 PMC3173284

[62] Herrero R Espinoza M Molinari G Etchemendy E Garcia-Palacios A Botella C . Psychometric properties of the general self Efficacy-12 scale in Spanish: general and clinical population samples. Compr Psychiatry. (2014) 55:1738–43. doi: 10.1016/j.comppsych.2014.05.015, 24973225

[63] Rosenberg M. Society and the Adolescent Self-Image. Princeton: Princeton University Press (1965).

[64] Martín-Albo J Núñez JL Navarro JG Grijalvo F. The Rosenberg self-esteem scale: translation and validation in university students. Span J Psychol. (2007) 10:458–67. doi: 10.1017/S1138741600006727, 17992972

[65] Moreno B Alonso M Álvaréz E. Sense of coherence, resistant, personality, self-esteem and health. J Health Psychol. (1997) 9:115–37.

[66] Hayes AF Cai L. Using heteroskedasticity-consistent standard error estimators in OLS regression: an introduction and software implementation. Behav Res Methods. (2007) 39:709–22. doi: 10.3758/BF03192961, 18183883

[67] Hayes A. Introduction to Mediation, Moderation, and Conditional Process Analysis. A Regression-Based Approach. 3rd ed. New York, NY: Guildford press (2022). p. 732.

[68] IBM Corp. IBM SPSS Statistics for Windows, Version 25.0 (2017) Available online at: https://www.ibm.com/ (Accessed July 10, 2025).

[69] Stewart DW Adams CE Cano MA Correa-Fernández V Li Y Waters AJ . Associations between health literacy and established predictors of smoking cessation. Am J Public Health. (2013) 103:e43–9. doi: 10.2105/AJPH.2012.301062, 23678912 PMC3682601

[70] Ramsey MW Chen-Sankey JC Reese-Smith J Choi K. Association between marital status and cigarette smoking: variation by race and ethnicity. Prev Med. (2019) 119:48–51. doi: 10.1016/j.ypmed.2018.12.010, 30576684 PMC6995657

[71] Greene J Hibbard JH. Why Does patient activation matter? An examination of the relationships between patient activation and health-related outcomes. J Gen Intern Med. (2012) 27:520–6. doi: 10.1007/s11606-011-1931-2, 22127797 PMC3326094

[72] Wilhite JA Velcani F Watsula-Morley A Hanley K Altshuler L Kalet A . Igniting activation: using unannounced standardized patients to measure patient activation in smoking cessation. Addict Behav Rep. (2019) 9:100179. doi: 10.1016/j.abrep.2019.100179, 31193839 PMC6544561

[73] Panahi R Hosseini N Ramezankhani A Anbari M Amjadian M Dehghankar L . Measuring the structures of the health belief model integrated with health literacy in predicting university students’ adoption of smoking preventive behaviors. J Prev Med Hyg. (2022) 63:E51–8. doi: 10.15167/2421-4248/JPMH2022.63.1.2236, 35647364 PMC9121676

[74] Rababah JA Al-Hammouri MM. Health literacy and smoking habits among a sample of Jordanian university students. J Community Health. (2023) 48:30–7. doi: 10.1007/s10900-022-01139-8, 36107378

[75] Li M Sonoda N Koh C Yasumoto R Morimoto A. Meta-analysis of the association between health literacy and smoking. Popul Med. (2022) 4:1–11. doi: 10.18332/popmed/152572

[76] Al Thani M Leventakou V Sofroniou A Butt HI Hakim IA Thomson C . Factors associated with baseline smoking self-efficacy among male Qatari residents enrolled in a quit smoking study. PLoS One. (2022) 17:e0263306. doi: 10.1371/journal.pone.0263306, 35085368 PMC8794180

[77] Rabani Bavojdan M Towhidi A Rahmati A. The relationship between mental health and general self-efficacy beliefs, coping strategies and locus of control in male drug abusers. Addict Health. (2011) 3:111–8. 24494125 PMC3905536

[78] Jamal M Willem Van der Does AJ Cuijpers P Penninx BW. Association of smoking and nicotine dependence with severity and course of symptoms in patients with depressive or anxiety disorder. Drug Alcohol Depend. (2012) 126:138–46. doi: 10.1016/j.drugalcdep.2012.05.001 22633368

[79] Méndez-López F Oliván-Blázquez B Domínguez García M López-Del-Hoyo Y Tamayo-Morales O Magallón-Botaya R. Depressive and anxious symptoms increase with problematic technologies use among adults: the effects of personal factors related to health behavior. Psychol Res Behav Manag. (2023) 16:2499–515. doi: 10.2147/PRBM.S412013, 37426389 PMC10329434

[80] Dahlgren G Whitehead M. The Dahlgren-Whitehead model of health determinants: 30 years on and still chasing rainbows. Public Health. (2021) 199:20–4. doi: 10.1016/j.puhe.2021.08.009, 34534885

[81] Ozili PK. The acceptable R-square in empirical modelling for social science research. SSRN Electron J. (2022). 134–143. doi: 10.2139/ssrn.4128165

[82] World Health Organization Depressive disorder (2023) Available online at: https://www.who.int/news-room/fact-sheets/detail/depression (Accessed April 10, 2023)

[83] Álvarez-Hernández G Delgado-DelaMora J. Diseño de Estudios Epidemiológicos. I. El Estudio Transversal: Tomando una Fotografía de la Salud y la Enfermedad. Bol Clin Hosp Infant Edo Son. (2015) 32:26–34.

[84] Wang X Cheng Z. Cross-sectional studies. Chest. (2020) 158:S65–71. doi: 10.1016/j.chest.2020.03.012, 32658654

[85] Glass D Kelsall H Slegers C Forbes A Loff B Zion D . A telephone survey of factors affecting willingness to participate in health research surveys. BMC Public Health. (2015) 15:1017. doi: 10.1186/s12889-015-2350-9, 26438148 PMC4594742

[86] Choi B Granero R Pak A. Catálogo de sesgos o errores en cuestionarios sobre salud. Rev Costarr Salud Pública. (2010) 19:106–18.

[87] Demetriou C Ozer BU Essau CA. "Self-Report Questionnaires". In: The Encyclopedia of Clinical Psychology. Hoboken, NJ, USA: John Wiley & Sons, Inc. (2015). p. 1–6.

