# Supplementary material for: Depression and anxiety symptoms, health behavior–related personal factors, and tobacco smoking problem: a secondary data analysis
Source: Front Public Health. 2026 Jul 16;14:1886431. doi: 10.3389/fpubh.2026.1886431 (PMC13422414; doi:10.3389/fpubh.2026.1886431)
Supplement: Supplementary file 1 [file Data_sheet_1.docx]

**Supplementary Figure 1.** Flow Diagram following the Strengthening the Reporting of Observational studies in Epidemiology (STROBE) guidelines.


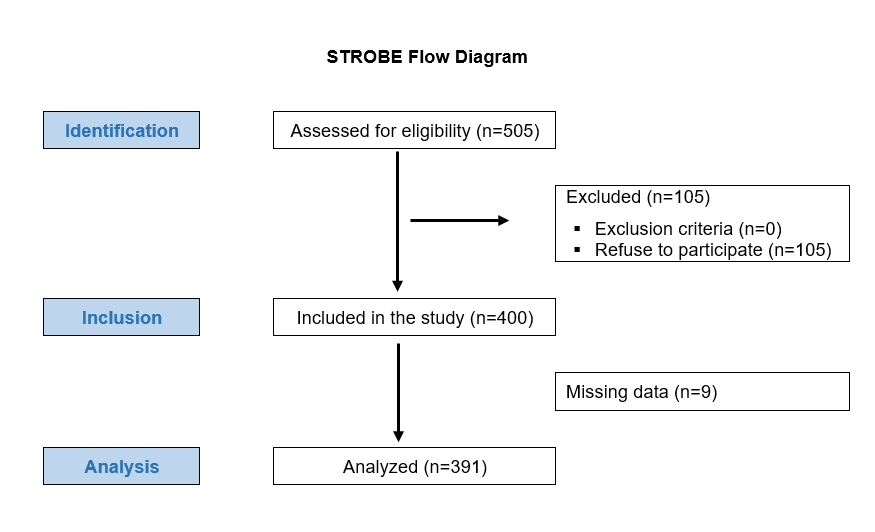


**Supplementary Table 1**. The mediating role of depression between personal factors and the presence of tobacco dependence.

| ***Model*** | **Estimate log Odds** | **95% C.I.** | | **p-value** |
| --- | --- | --- | --- | --- |
|  |  | **Lower** | **Upper** |  |
| **Indirect effects** |  |  |  |  |
| Self-efficacy ⇒ Depression ⇒ Presence of tobacco dependence | -0.00130 | -0.00270 | 9.55e-5 | 0.068 |
| Resilience ⇒ Depression ⇒ Presence of tobacco dependence | -3.15e−5 | -7.71e−4 | 7.08e-4 | 0.934 |
| Extraversion ⇒ Depression ⇒ Presence of tobacco dependence | 6.02e-4 | -0.00192 | 0.00312 | 0.639 |
| Agreeableness ⇒ Depression ⇒ Presence of tobacco dependence | 6.65e-4 | -0.00181 | 0.00314 | 0.599 |
| Conscientiousnes ⇒ Depression ⇒ Presence of tobacco dependence | 3.47e-4 | -0.00232 | 0.00301 | 0.799 |
| Neuroticism ⇒ Depression ⇒ Presence of tobacco dependence | -0.00435 | -0.00860 | 0.00306 | 0.505 |
| Openness ⇒ Depression ⇒ Presence of tobacco dependence | 9.55e-4 | -0.00185 | 0.00376 | 0.505 |
| Sense of coherence ⇒ Depression ⇒ Presence of tobacco dependence | -6.53e−4 | -0.00153 | 2.26e-4 | 0.145 |
| Self-esteem ⇒ Depression ⇒ Presence of tobacco dependence | -0.00175 | -0.00370 | 1.94e-4 | 0.078 |
|  |  |  |  |  |
| **Direct effects** |  |  |  |  |
| Self-efficacy ⇒ Presence of tobacco dependence | -0.00215 | -0.00950 | 0.00519 | 0.566 |
| Resilience ⇒ Presence of tobacco dependence | 0.00368 | -0.00266 | 0.01002 | 0.256 |
| Extraversion ⇒ Presence of tobacco dependence | -0.00440 | -0.02556 | 0.01675 | 0.683 |
| Agreeableness ⇒ Presence of tobacco dependence | -0.00229 | -0.02298 | 0.01841 | 0.829 |
| Conscientiousnes ⇒ Presence of tobacco dependence | 0.01173 | -0.01098 | 0.03445 | 0.311 |
| Neuroticism ⇒ Presence of tobacco dependence | -0.00832 | -0.02647 | 0.00984 | 0.369 |
| Openness ⇒ Presence of tobacco dependence | 0.00493 | -0.01812 | 0.02799 | 0.675 |
| Sense of coherence ⇒ Presence of tobacco dependence | -0.00207 | -0.00793 | 0.00379 | 0.490 |
| Self-esteem ⇒ Presence of tobacco dependence | -0.00436 | -0.01515 | 0.00643 | 0.428 |
|  |  |  |  |  |
| **Total effects** |  |  |  |  |
| Self-efficacy ⇒ Presence of tobacco dependence | -0.00345 | -0.01077 | 0.00387 | 0.355 |
| Resilience ⇒ Presence of tobacco dependence | 0.00364 | -0.00275 | 0.01004 | 0.264 |
| Extraversion ⇒ Presence of tobacco dependence | -0.00380 | -0.02512 | 0.01752 | 0.727 |
| Agreeableness ⇒ Presence of tobacco dependence | -0.00162 | -0.02247 | 0.01923 | 0.879 |
| Conscientiousnes ⇒ Presence of tobacco dependence | 0.01208 | -0.01081 | 0.03498 | 0.301 |
| Neuroticism ⇒ Presence of tobacco dependence | -0.01267 | -0.03058 | 0.00525 | 0.166 |
| Openness ⇒ Presence of tobacco dependence | 0.00589 | -0.01734 | 0.02912 | 0.619 |
| Sense of coherence ⇒ Presence of tobacco dependence | -0.00272 | -0.00860 | 0.00316 | 0.365 |
| Self-esteem ⇒ Presence of tobacco dependence | -0.00612 | -0.01689 | 0.00466 | 0.266 |

**Supplementary Table 2**.The mediating role of depression between personal factors and cigarettes consumed per day.

| ***Model*** | **Estimate** | **95 % C.I** | | **β** | **p-values** |
| --- | --- | --- | --- | --- | --- |
|  |  | **Lower** | **Upper** |  |  |
| **Indirect effects** |  |  |  |  |  |
| Self-efficacy ⇒ Depression ⇒ Cigarettes consumed per day | -0.02755 | -0.0566 | 0.00151 | -0.02167 | 0.063 |
| Resilience ⇒ Depression ⇒ Cigarettes consumed per day | -6.67e−4 | -0.0163 | 0.01501 | -6.05e−4 | 0.934 |
| Extraversion ⇒ Depression ⇒ Cigarettes consumed per day | 0.01276 | -0.0406 | 0.06607 | 0.00305 | 0.639 |
| Agreeableness ⇒ Depression ⇒ Cigarettes consumed per day | 0.01409 | -0.0383 | 0.06651 | 0.00338 | 0.598 |
| Conscientiousnes ⇒ Depression ⇒ Cigarettes consumed per day | 0.00734 | -0.0491 | 0.06378 | 0.00156 | 0.799 |
| Neuroticism ⇒ Depression ⇒ Cigarettes consumed per day | -0.09215 | -0.1803 | -0.00398 | 0.00408 | 0.504 |
| Openness ⇒ Depression ⇒ Cigarettes consumed per day | 0.02023 | -0.0391 | 0.07958 | 0.00438 | 0.504 |
| Sense of coherence ⇒ Depression ⇒ Cigarettes consumed per day | -0.01384 | -0.0323 | 0.00459 | -0.01244 | 0.141 |
| Self-esteem ⇒ Depression ⇒ Cigarettes consumed per day | -0.03714 | -0.0777 | 0.00344 | -0.01947 | 0.073 |
|  |  |  |  |  |  |
| **Direct effects** |  |  |  |  |  |
| Self-efficacy ⇒ Cigarettes consumed per day | -0.06142 | -0.2128 | 0.08993 | -0.04832 | 0.426 |
| Resilience ⇒ Cigarettes consumed per day | 0.04725 | -0.0834 | 0.17788 | 0.04291 | 0.478 |
| Extraversion ⇒ Cigarettes consumed per day | 0.03075 | -0.4051 | 0.46662 | 0.00736 | 0.890 |
| Agreeableness ⇒ Cigarettes consumed per day | -0.00629 | -0.4326 | 0.42005 | -0.00151 | 0.977 |
| Conscientiousnes ⇒ Cigarettes consumed per day | 0.16597 | -0.3020 | 0.63396 | 0.03526 | 0.487 |
| Neuroticism ⇒ Cigarettes consumed per day | -0.00707 | -0.3811 | 0.36697 | -0.00197 | 0.970 |
| Openness ⇒ Cigarettes consumed per day | 0.21258 | -0.2625 | 0.68763 | 0.04608 | 0.380 |
| Sense of coherence ⇒ Cigarettes consumed per day | -0.08679 | -0.2075 | 0.03394 | -0.07805 | 0.159 |
| Self-esteem ⇒ Cigarettes consumed per day | -0.04455 | -0.2668 | 0.17775 | -0.02335 | 0.694 |
|  |  |  |  |  |  |
| **Total effects** |  |  |  |  |  |
| Self-efficacy ⇒ Cigarettes consumed per day | -0.08897 | -0.2398 | 0.06191 | -0.06999 | 0.248 |
| Resilience ⇒ Cigarettes consumed per day | 0.04658 | -0.0851 | 0.17831 | 0.04231 | 0.488 |
| Extraversion ⇒ Cigarettes consumed per day | 0.04351 | -0.3959 | 0.48294 | 0.01041 | 0.846 |
| Agreeableness ⇒ Cigarettes consumed per day | 0.00780 | -0.4220 | 0.43759 | 0.00187 | 0.972 |
| Conscientiousnes ⇒ Cigarettes consumed per day | 0.17332 | -0.2986 | 0.64522 | 0.03682 | 0.472 |
| Neuroticism ⇒ Cigarettes consumed per day | -0.09922 | -0.4685 | 0.27002 | -0.02769 | 0.598 |
| Openness ⇒ Cigarettes consumed per day | 0.23280 | -0.2460 | 0.71158 | 0.05046 | 0.341 |
| Sense of coherence ⇒ Cigarettes consumed per day | -0.10062 | -0.2218 | 0.02057 | -0.09049 | 0.104 |
| Self-esteem ⇒ Cigarettes consumed per day | -0.08168 | -0.3037 | 0.14033 | -0.04282 | 0.471 |

**Supplementary Table 3.** The mediating role of anxiety between personal factors and the presence of tobacco dependence.

| ***Model*** | **Estimate log Odds** | **95% C.I.** | | **p-value** |
| --- | --- | --- | --- | --- |
|  |  | **Lower** | **Upper** |  |
| **Indirect effects** |  |  |  |  |
| Self-efficacy ⇒ Anxiety ⇒ Presence of tobacco dependence | -2.70e−4 | -0.00126 | 7.19e-4 | 0.593 |
| Resilience ⇒ Anxiety ⇒ Presence of tobacco dependence | 1.86e-5 | -1.69e−4 | 2.06e-4 | 0.846 |
| Health literacy⇒ Anxiety ⇒ Presence of tobacco dependence | 5.09e-4 | -0.00139 | 0.00241 | 0.600 |
| Extraversion ⇒ Anxiety ⇒ Presence of tobacco dependence | -3.43e−4 | -0.00171 | 0.00102 | 0.622 |
| Activation⇒ Anxiety ⇒ Presence of tobacco dependence | -6.65e−5 | -3.14e−4 | 1.81e-4 | 0.599 |
| Agreeableness ⇒ Anxiety ⇒ Presence of tobacco dependence | 4.29e-4 | -0.00121 | 0.00207 | 0.609 |
| Conscientiousness ⇒ Anxiety ⇒ Presence of tobacco dependence | 1.43e-4 | -6.69e−4 | 9.54e-4 | 0.730 |
| Neuroticism ⇒ Anxiety ⇒ Presence of tobacco dependence | -0.00157 | -0.00722 | 0.00409 | 0.587 |
| Openness ⇒ Anxiety ⇒ Presence of tobacco dependence | 7.30e-5 | -6.17e−4 | 7.63e-4 | 0.836 |
| Sense of coherence ⇒ Anxiety ⇒ Presence of tobacco dependence | 1.64e-5 | -1.55e−4 | 1.88e-4 | 0.851 |
| Self-esteem ⇒ Anxiety ⇒ Presence of tobacco dependence | -3.92e−4 | -0.00183 | 0.00105 | 0.593 |
|  |  |  |  |  |
| **Direct effects** |  |  |  |  |
| Self-efficacy ⇒ Presence of tobacco dependence | -0.00208 | -0.00940 | 0.00525 | 0.578 |
| Resilience ⇒ Presence of tobacco dependence | 0.00422 | -0.00210 | 0.01054 | 0.191 |
| Health literacy⇒ Presence of tobacco dependence | -0.01568 | -0.03475 | 0.00339 | 0.107 |
| Extraversion ⇒ Presence of tobacco dependence | -0.00383 | -0.02489 | 0.01722 | 0.721 |
| Activation⇒ Presence of tobacco dependence | -0.00237 | -0.00474 | -5.05e−6 | 0.050 |
| Agreeableness ⇒ Presence of tobacco dependence | -0.00341 | -0.02410 | 0.01728 | 0.747 |
| Conscientiousness ⇒ Presence of tobacco dependence | 0.00978 | -0.01292 | 0.03247 | 0.398 |
| Neuroticism ⇒ Presence of tobacco dependence | -0.01062 | -0.02916 | 0.00793 | 0.262 |
| Openness ⇒ Presence of tobacco dependence | 0.00383 | -0.01922 | 0.02688 | 0.745 |
| Sense of coherence ⇒ Presence of tobacco dependence | -0.00222 | -0.00803 | 0.00359 | 0.454 |
| Self-esteem ⇒ Presence of tobacco dependence | -0.00450 | -0.01521 | 0.00622 | 0.411 |
|  |  |  |  |  |
| **Total effects** |  |  |  |  |
| Self-efficacy ⇒ Presence of tobacco dependence | -0.00235 | -0.00962 | 0.00493 | 0.527 |
| Resilience ⇒ Presence of tobacco dependence | 0.00424 | -0.00209 | 0.01057 | 0.190 |
| Health literacy⇒ Presence of tobacco dependence | -0.01517 | -0.03418 | 0.00384 | 0.118 |
| Extraversion ⇒ Presence of tobacco dependence | -0.00418 | -0.02523 | 0.01688 | 0.697 |
| Activation⇒ Presence of tobacco dependence | -0.00244 | -0.00480 | -7.97e−5 | 0.043 |
| Agreeableness ⇒ Presence of tobacco dependence | -0.00298 | -0.02365 | 0.01768 | 0.777 |
| Conscientiousness ⇒ Presence of tobacco dependence | 0.00992 | -0.01281 | 0.03265 | 0.392 |
| Neuroticism ⇒ Presence of tobacco dependence | -0.01218 | -0.02988 | 0.00551 | 0.177 |
| Openness ⇒ Presence of tobacco dependence | 0.00391 | -0.01918 | 0.02699 | 0.740 |
| Sense of coherence ⇒ Presence of tobacco dependence | -0.00220 | -0.00802 | 0.00361 | 0.458 |
| Self-esteem ⇒ Presence of tobacco dependence | -0.00489 | -0.01553 | 0.00575 | 0.368 |

**Supplementary Table 4.** The mediating role of anxiety between personal factors and cigarettes consumed per day.

| ***Model*** | **Estimate** | **95% C.I. (a)** | | **β** | **p-value** |
| --- | --- | --- | --- | --- | --- |
|  |  | **Lower** | **Upper** |  |  |
| **Indirect effects** |  |  |  |  |  |
| Self-efficacy ⇒ Anxiety ⇒ Cigarettes consumed per day | -0.00334 | -0.02333 | 0.01666 | -0.00262 | 0.744 |
| Resilience ⇒ Anxiety ⇒ Cigarettes consumed per day | 2.30e-4 | -0.00233 | 0.00279 | 2.09e-4 | 0.860 |
| Health literacy⇒ Anxiety ⇒ Cigarettes consumed per day | 0.00630 | -0.03175 | 0.04436 | 0.00173 | 0.745 |
| Extraversion ⇒ Anxiety ⇒ Cigarettes consumed per day | -0.00424 | -0.03050 | 0.02201 | -0.00102 | 0.751 |
| Activation⇒ Anxiety ⇒ Cigarettes consumed per day | -8.23e−4 | -0.00578 | 0.00414 | -0.00175 | 0.745 |
| Agreeableness ⇒ Anxiety ⇒ Cigarettes consumed per day | 0.00531 | -0.02705 | 0.03767 | 0.00127 | 0.748 |
| Conscientiousness ⇒ Anxiety ⇒ Cigarettes consumed per day | 0.00177 | -0.01131 | 0.01485 | 3.76e-4 | 0.791 |
| Neuroticism ⇒ Anxiety ⇒ Cigarettes consumed per day | -0.01941 | -0.13498 | 0.09616 | -0.00542 | 0.742 |
| Openness ⇒ Anxiety ⇒ Cigarettes consumed per day | 9.04e-4 | -0.00865 | 0.01046 | 1.96e-4 | 0.853 |
| Sense of coherence ⇒ Anxiety ⇒ Cigarettes consumed per day | 2.03e-4 | -0.00212 | 0.00253 | 1.82e-4 | 0.865 |
| Self-esteem ⇒ Anxiety ⇒ Cigarettes consumed per day | -0.00485 | -0.03392 | 0.02422 | -0.00254 | 0.744 |
|  |  |  |  |  |  |
| **Direct effects** |  |  |  |  |  |
| Self-efficacy ⇒ Cigarettes consumed per day | -0.05711 | -0.20718 | 0.09296 | -0.04493 | 0.456 |
| Resilience ⇒ Cigarettes consumed per day | 0.05995 | -0.06951 | 0.18942 | 0.05445 | 0.364 |
| Health literacy⇒ Cigarettes consumed per day | -0.36341 | -0.75406 | 0.02724 | -0.09966 | 0.068 |
| Extraversion ⇒ Cigarettes consumed per day | 0.03806 | -0.39331 | 0.46942 | 0.00911 | 0.863 |
| Activation⇒ Cigarettes consumed per day | -0.06521 | -0.11373 | -0.01668 | -0.13833 | 0.008 |
| Agreeableness ⇒ Cigarettes consumed per day | -0.03400 | -0.45780 | 0.38980 | -0.00816 | 0.875 |
| Conscientiousness ⇒ Cigarettes consumed per day | 0.11344 | -0.35147 | 0.57835 | 0.02410 | 0.632 |
| Neuroticism ⇒ Cigarettes consumed per day | -0.06802 | -0.44789 | 0.31184 | -0.01898 | 0.726 |
| Openness ⇒ Cigarettes consumed per day | 0.18360 | -0.28860 | 0.65581 | 0.03980 | 0.446 |
| Sense of coherence ⇒ Cigarettes consumed per day | -0.08814 | -0.20717 | 0.03088 | -0.07927 | 0.147 |
| Self-esteem ⇒ Cigarettes consumed per day | -0.04809 | -0.26764 | 0.17147 | -0.02521 | 0.668 |
|  |  |  |  |  |  |
| **Total effects** |  |  |  |  |  |
| Self-efficacy ⇒ Cigarettes consumed per day | -0.06045 | -0.20941 | 0.08852 | -0.04756 | 0.426 |
| Resilience ⇒ Cigarettes consumed per day | 0.06018 | -0.06946 | 0.18983 | 0.05466 | 0.363 |
| Health literacy⇒ Cigarettes consumed per day | -0.35710 | -0.74650 | 0.03229 | -0.09793 | 0.072 |
| Extraversion ⇒ Cigarettes consumed per day | 0.03381 | -0.39743 | 0.46505 | 0.00809 | 0.878 |
| Activation⇒ Cigarettes consumed per day | -0.06603 | -0.11438 | -0.01769 | -0.14008 | 0.007 |
| Agreeableness ⇒ Cigarettes consumed per day | -0.02869 | -0.45191 | 0.39454 | -0.00688 | 0.894 |
| Conscientiousness ⇒ Cigarettes consumed per day | 0.11521 | -0.35025 | 0.58066 | 0.02448 | 0.628 |
| Neuroticism ⇒ Cigarettes consumed per day | -0.08743 | -0.44986 | 0.27500 | -0.02440 | 0.636 |
| Openness ⇒ Cigarettes consumed per day | 0.18450 | -0.28835 | 0.65735 | 0.03999 | 0.444 |
| Sense of coherence ⇒ Cigarettes consumed per day | -0.08794 | -0.20713 | 0.03125 | -0.07909 | 0.148 |
| Self-esteem ⇒ Cigarettes consumed per day | -0.05294 | -0.27090 | 0.16503 | -0.02775 | 0.634 |
